# Self-Etching-Induced Morphological Evolution of ZnO Microrods Grown on FTO Glass by Hydrothermal Method

**DOI:** 10.1186/s11671-015-1140-8

**Published:** 2015-10-30

**Authors:** Wen-Dung Hsu, Jenn-Kai Tsai, Teen-Hang Meen, Tian-Chiuan Wu, Yan-Kuan He, Yu-Da Lai

**Affiliations:** Department of Materials Science and Engineering, National Cheng Kung University, Tainan, 70101 Taiwan; Department of Electronic Engineering, National Formosa University, Yunlin, 632 Taiwan

**Keywords:** Hydrothermal method, ZnO, Microrod, Micropencil, ODS, HMT, Zn(NO_3_)_2_·6H_2_O

## Abstract

In this research, the zinc oxide (ZnO) microrods were grown by hydrothermal method on fluorine-doped tin oxide (FTO) glass functionalized by self-assembled monolayer of octadecyltrimethoxysilane (ODS; CH_3_(CH_2_)_17_Si(OCH_3_)_3_). The sharp-tip or polygonal shape with specific facets at the top end of ZnO microrods can be obtained by post retention at low temperature. The morphologies were characterized by the field-emission scanning electron microscope (FESEM) and transmission electron microscopy (TEM). The results confirm that the morphology change at the top end is due to self-etching. The mechanism responsible for the formation of various top-end morphologies was proposed. The specific facets that left after 6-h retention were identified. The room-temperature micro-photoluminescence spectra showed a strong ultraviolet emission at 387 nm, and a broad emission at a range of from 500 to 700 nm. The morphology change also influences the photoluminescence (PL) spectra. A satellite peak in the UV emission spectra was observed. The peak may be attributed to the morphology effect of the microrods.

## Background

In recent years, controlling the morphology and size of ZnO nanomaterials has attracted intensive attentions. ZnO is one of II–VI semiconductors with a direct band gap of 3.37 eV. It has excellent chemical stability. The optical, electrical, and other physicochemical properties [1–4] of ZnO nanomaterials can be tuned by changing its morphology, thus it has been widely applied in many promising devices such as optoelectronics [5, 6], field emission arrays [7, 8], sensors [9], light-emitting devices [10], solar cells [11, 12], and memory devices [13]. ZnO nanomaterials have various morphologies, such as nanotubes, nanowires, nanorods, nanopyramids, and nanopins, depending on the synthesis method. Different ZnO structures can be applied to different fields, therefore various synthesis methods, such as chemical vapor deposition [14, 15], vapor transport deposition [16], magnetron sputtering method [17], and hydrothermal method [10, 18] have been reported to produce different ZnO nanostructures. Steadily producing morphology controllable ZnO nanomaterials by cost-efficient methods, however, is still a challenge. Hydrothermal method is one of highly promising methods to fulfill the demand because of its low synthesis temperature, low cost, less complicated technique, and good potential for scale-up.

In this study, effect of low temperature retention after growth of ZnO microrods at 90 °C for 12 h was investigated. The retention means that the ZnO microrods were kept at 45 °C for several hours immediately after the growth. X-ray diffraction (XRD) pattern was used to study the crystallography of ZnO microrods. SEM and TEM were adopted to identify the morphology of ZnO microrods. UV emission spectra were used to characterize optical properties of ZnO microrods. The possible mechanism that is responsible for the observed morphology was proposed.

## Methods

### Fabrication of ZnO Microstructures

**Fig. 1:**
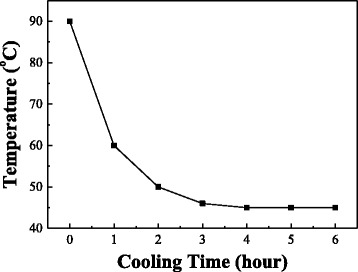
The real temperature of reaction solution during retention

Transparent fluorine-doped tin oxide (FTO) conductive glass was cleaned by acetone, methanol, and deionized water in ultrasonic oscillator to remove contaminations on surfaces. The FTO substrate was then put into Teflon beaker and thermal evaporated 0.2 ml octadecyltrimethoxysilane (ODS; CH_3_(CH_2_)_17_Si(OCH_3_)_3_, Acros, Geel, Belgium) by using autoclave at 150 °C for 1 h. After that, an ODS-treated surface of FTO glass substrate was obtained. The purpose of this treatment is to enhance the following growth of ZnO microrods. The aqueous solutions composed of 0.06 M of zinc nitrate hexahydrate (Zn(NO_3_)_2_·6H_2_O, Alfa Aesar, Ward Hill, MA, USA) and 0.03 M of hexamethylenetetramine (CH_2_)_6_N_4_; HMT, Hayashi Pure Chemical Industry Co., Ltd, Osaka, Japan) were mixed in a rectangle Teflon beaker. The solution was designed to be a zinc-rich ambience. The FTO glass was put into the rectangular Teflon beaker vertically at temperatures 90 °C for 12 h. After 12 h, the beaker was moved to 45 °C environment for 0 to 6 h to study the effect of retention. The temperature of the solution during retention as a function of time is shown in Fig. 1. At last, the samples were taken out from the beaker, then subsequently rinsed with deionized water to remove residual chemicals and finally baked at 150 °C for 1 h to remove water.

### Characterizations

The morphology of ZnO microrods was examined by scanning electron microscope (SEM; Seiko Instruments Inc., SII, Chiba, Chiba Prefecture, Japan). The detailed structure was examined by transmission electron microscopy (TEM; Tecnai G2, FEI, Hillsboro, OR, USA). The crystal phase and crystallinity were analyzed by X-ray diffraction (XRD; D1, Jordan Valley Semiconductors Ltd., Austin, TX, USA). The photoluminescence (PL; HR800, HORIBA Scientific, Kyoto, Japan) was used to characterize the optical properties of the ZnO microrod arrays at room temperature. The light source was He–Cd laser with wavelength of 325 nm.

## Results and Discussion

**Fig. 2:**
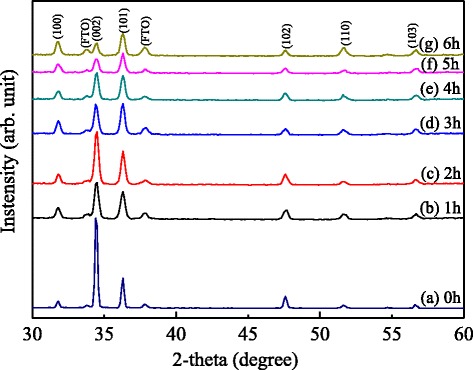
XRD patterns of ZnO microrods synthesized on FTO glass with a retention time from 0 to 6 h

Figure 2 shows XRD patterns of all the samples after retention from 0 to 6 h. The three main diffraction peaks at 2*θ* = 31.79°, 34.50°, and 36.29° come from the (10–10), (0002), and (10–11) diffraction pattern of hexagonal wurtzite structure, respectively. They match well with the JCPDS card of no. 36-1451 with lattice constants of *a* = 3.25 and *c* = 5.21 Å. The diffraction peaks of FTO glass also can be observed indicating low area density ZnO microrods were grown on functionalized FTO glass because no seed layer was used. The intensity ratio of (0002) to (10–11) peaks is strong for the sample without retention. While the retention time increases, the ratio decreases.

**Fig. 3:**
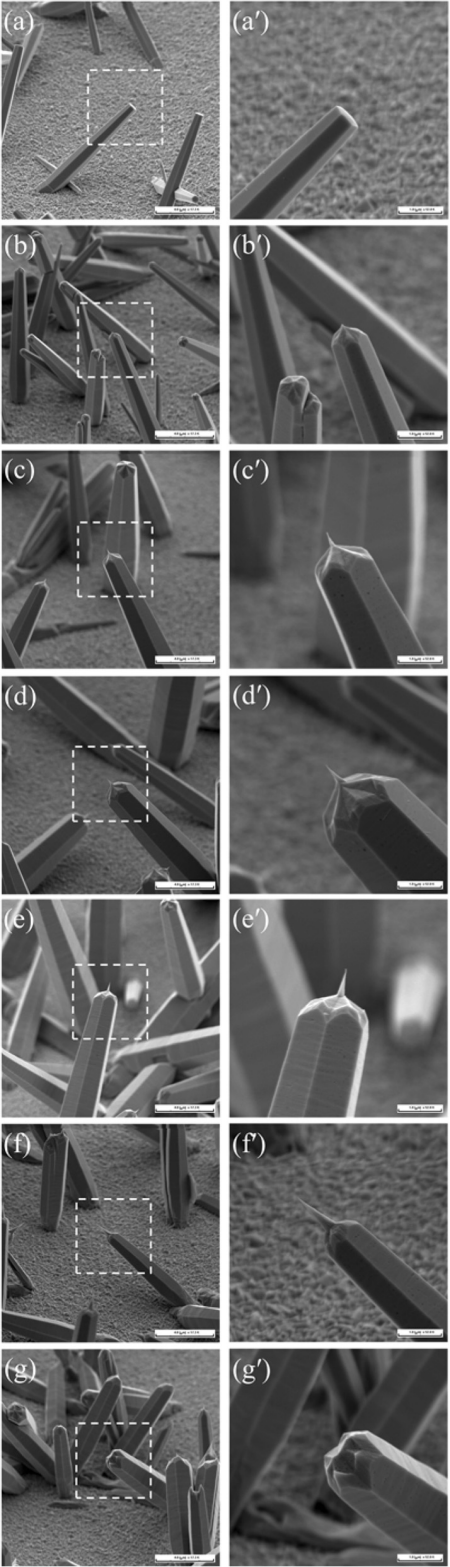
Low and high magnificent 55° tilt-view FESEM images of ZnO microrods grown on FTO glass after 0- to 6-h retention. **a**, **a'** 0-h retention. **b**, **b'** 1-h retention. **c**, **c'** 2-h retention. **d**, **d'** 3-h retention. **e**, **e'** 4-h retention. **f**, **f'** 5-h retention. **g**, **g'** 6-h retention

Figure 3 shows 55° tilt-view SEM images of ZnO microrods grown on functionalized FTO glass surface. The square portion marked by dash line in the left-side images is further magnified and show in the corresponding right-side images. The right-side images shown as Fig. 3a–g indicate that the area density of microrods is low because a seed-free substrate was employed. The result is consistent with the FTO substrate diffraction pattern observed in XRD data. Figure 3a, a' shows the well-defined hexagonal structure of ZnO microrods grown at 90 °C for 12 h by hydrothermal method with no post retention. After 1-h retention, the flat top end becomes a hexagonal cone shape as shown in Fig. 3b, b'. When the retention time increases, the hexagonal cone top end becomes a sharp-tip shape as shown in Fig. 3c–f and c'–f'. The length of the tip increases with the increase of retention time. The tip then disappears after 6-h retention as shown in Fig. 3g, g'. At this stage, the top end shows a polygon cone shape formed by specific facets. In Fig. 3b, b', “fly over” attachment of two microrods is also observed. The same structure was reported by B. Liu et al. [19].

**Fig. 4:**
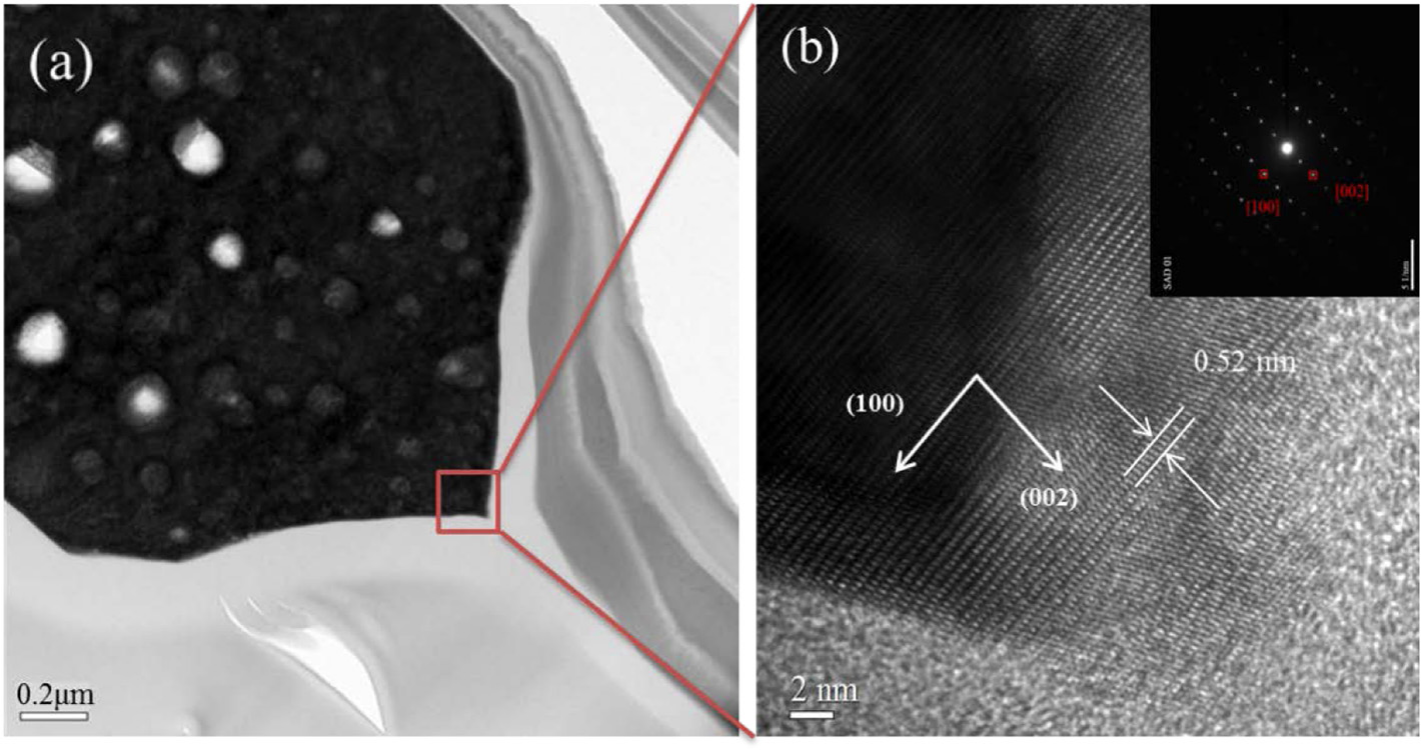
**a** Low-magnification and **b** high-resolution TEM images of ZnO tip at the top end of microrods grown by hydrothermal method with 3-h retention. The *inset* of (**b**) shows the corresponding SAED pattern

Figure 4 shows TEM image of the ZnO microrods after 3-h retention. The sample was prepared by focus ion beam instrument. Some part of the sharp tip was cut away during ion beam bombardment. Figure 4a shows the low-magnification image, which is consistent with the FESEM images shown in Fig 3d, d'. The high-resolution TEM (HRTEM) image of the same sample at the tip area is shown in Fig. 4b. The HRTEM image clearly reveals a distance of 0.52 nm between two lattice fringes that coincide with the distance between two (0001) planes of the hexagonal wurtzite ZnO. The corresponding selected area electron diffraction (SAED) pattern shown in the inset of Fig. 4b confirms the results of HRTEM. The outcome indicates that the ZnO tip is single crystalline.

**Fig. 5:**
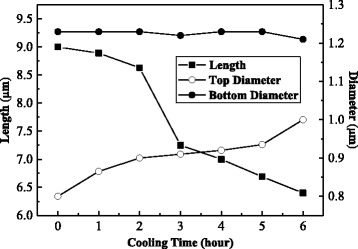
Length and diameters of top end and bottom of ZnO microrods at various retention times

Figure 5 shows the length and diameters of top end and bottom of ZnO microrods at the retention time 0–6 h. The detailed numbers are listed in Table 1. When the retention time increases, the length of ZnO microrods decreases; however, the bottom diameter and the top-end diameter changes very little. Thus, retention causes shortening of microrods indicating that ZnO microrods dissolve back into the solution. In other words, etching, instead of growth, is the dominant process during retention and the tip formed at the top end is the result of etching. The evidence also can be found by that the ZnO tip is a single crystalline structure as its rod body shown in Fig. 4b.

**Table 1:** The UV emission spectra of a single ZnO microstructure and length and diameters of top end and bottom of the ZnO microstructure at different cooling times

Product (h)	PL peak1 (eV)	PL peak2 (eV)	Intensity ratio (peak2/peak1)	Length (μm)	Top-end diameter (μm)	Bottom diameter (μm)
1	3.22	3.29	0.216	8.89	0.80	1.23
2	3.20	3.32	0.331	8.63	0.86	1.23
3	3.20	3.32	0.251	7.25	0.90	1.22
4	3.20	3.32	0.364	7.00	0.91	1.23
5	3.20	3.32	0.187	6.69	0.92	1.23
6	3.21	3.30	0.490	6.49	0.93	1.12

*PL* photoluminescence

**Fig. 6:**
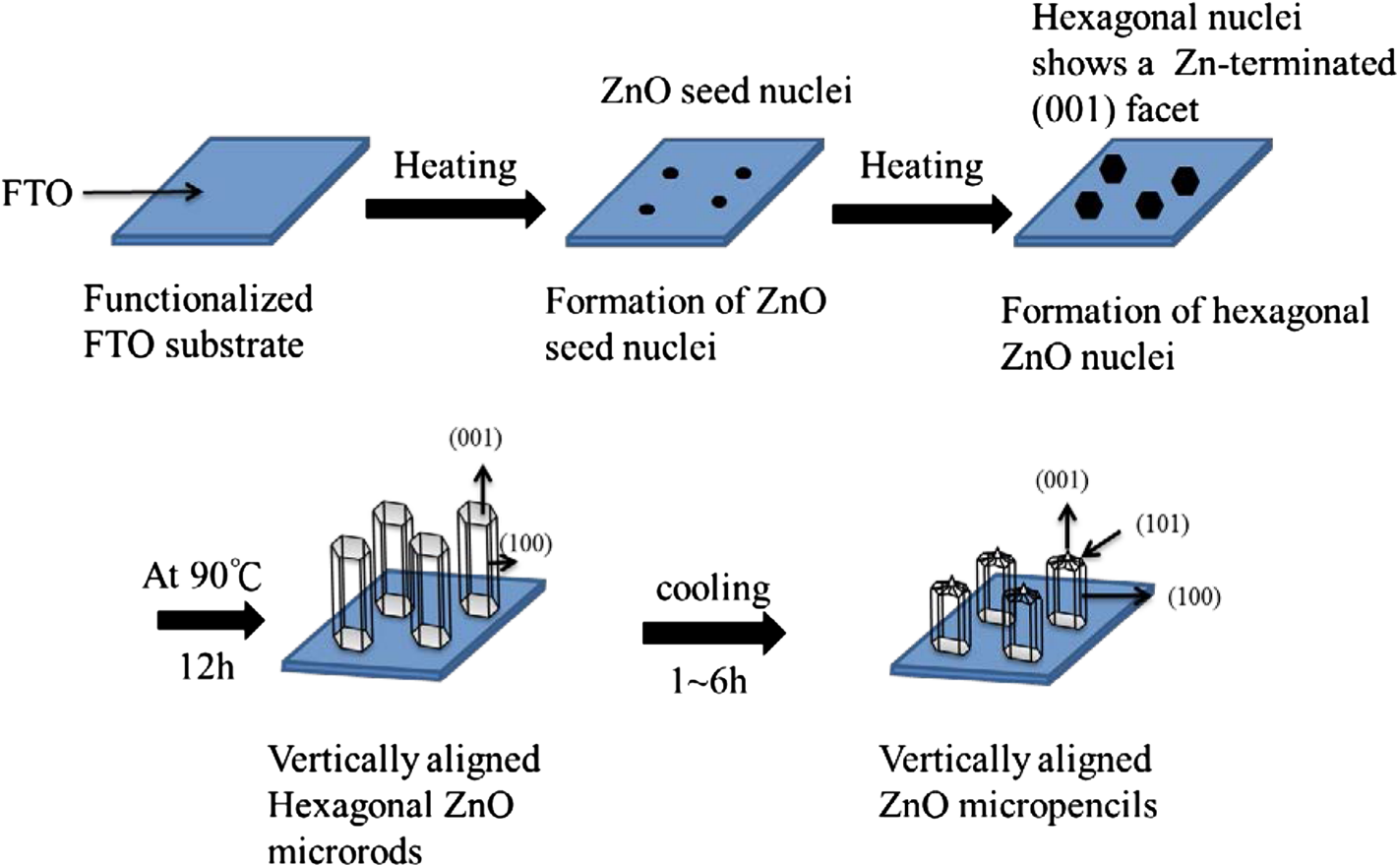
Schematic illustration of the growth and etching mechanism for the formation of ZnO microrods with a sharp tip on the top end after an appropriate retention time

On the basis of the experimental results, the etching mechanism of ZnO microrods is proposed. Figure 6 illustrates the etching process of ZnO microrods to form a sharp-tip structure at the top ends. The chemical reactions for ZnO microrod growth can be described as Eq. (1)–(4) [20]:

1 $$ {\mathrm{C}}_6{\mathrm{H}}_{12}{\mathrm{N}}_4+6{\mathrm{H}}_2\mathrm{O}\to\ 6{\mathrm{C}\mathrm{H}}_2\mathrm{O} + 4{\mathrm{N}\mathrm{H}}_3 $$

2 $$ {\mathrm{NH}}_3 + {\mathrm{H}}_2\mathrm{O}\leftrightarrow {{\mathrm{NH}}_4}^{+} + {\mathrm{OH}}^{-} $$

3 $$ {\mathrm{Zn}}^{2+} + 2{\mathrm{OH}}^{-}\to \mathrm{Z}\mathrm{n}{\left(\mathrm{O}\mathrm{H}\right)}_2 $$

4 $$ \mathrm{Z}\mathrm{n}{\left(\mathrm{O}\mathrm{H}\right)}_2\leftrightarrow \mathrm{Z}\mathrm{n}\mathrm{O}+{\mathrm{H}}_2\mathrm{O} $$

Initially, in the reaction solution, the Zn(NO_3_)_2_ dissolves in the solution and provides Zn^2+^ ions. Hydrolysis of HMT and the subsequent reaction with water generate OH^−^ shown as Eq. (1)–(2). As the concentration of Zn^2+^ and OH^−^ ions exceeds a critical value, the precipitation of Zn(OH)_2_ starts (Eq. (3)). Then the hydrolysis of Zn(OH)_2_ produces ZnO molecules (Eq. (4)). Aggregation of ZnO molecules would form ZnO nuclei. ZnO nuclei are the building blocks for growth of ZnO microrods. Due to crystal habits of ZnO, the nuclei have a hexagonal shape. In the hexagonal wurtzite phase, the ZnO has polar and non-polar faces. In polar ZnO crystals, the zinc and oxygen atoms are arranged alternately along the *c*-axis, and the top-end surfaces are Zn-terminated (0001) and are catalytically active, while the bottom surfaces are O-terminated (000-1) and are chemically inert [21]. As an amphoteric oxide, ZnO can react with both H^+^ and OH^−^ ions, and the products are soluble salts. Thus, as the pH value changes in the solution due to temperature changing, selective etching of ZnO microrods forms the sharp tip on the top end.

The etching started from the corner of top-end hexagonal surface of microrods and in overall eroded downward along [0001] axis. The etching rate is proportional to stability of surface that can be quantified by surface energy. Ions at the corner of the top-end hexagonal surface have the fewest coordination numbers, hence are the most unstable spots and are expected to dissolve back (etched) first [22]. For the retention time from 0–5 h, the etching rate is high. The tip shape formed due to the fast etching from the corners. For the retention time after 5 h, the temperature is low and the stable facets are shown.

In order to identify the facets of the remaining surfaces, the six smooth prismatic side planes of the rods are identified according to K. S. Chen et al. [23]. They used H implantation and subsequent high temperature annealing on ZnO surface to determine the relative surface formation energies and to construct a Wulff plot. Their results indicate that the six smooth prismatic side planes are (10$\overline{1}$0), (01$\overline{1}$0), ($\overline{1}$010), (0$\overline{1}$10), (1$\overline{1}$00), and ($\overline{1}$100), consistent with the reports from B. Liu et al. [19] and W. Zheng et al. [24]. The six smooth prismatic side planes intersect at six <1$\overline{2}$10> directions. Since the etching started from the edge of the corner of top-end hexagonal surface, the remaining surfaces would be those stable facets for the microrod with zone axis along six <1$\overline{2}$10>. Thus, in Fig. 3g, g', the 12 tilt surfaces can be divided into six groups belonging to six <1$\overline{2}$10> directions. Again K. S. Chen et al. [23] have reported the surface formation energy of ZnO nanowire with zone axis along [1$\overline{2}$10]. Their data show that the most stable facets are ($\overline{1}$014) and (10$\overline{1}$4). Therefore, 12 tilt surfaces are the ($\overline{1}$014) and (10$\overline{1}$4) facets of each [1$\overline{2}$10] directions as zone axis.

**Fig. 7:**
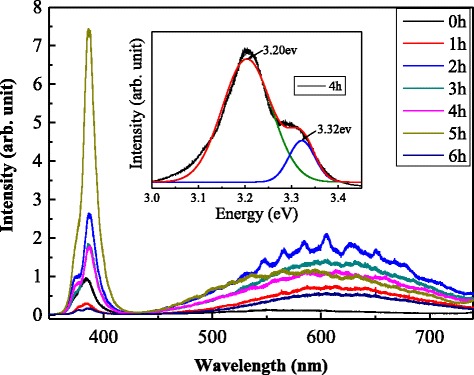
Room-temperature PL spectra of single ZnO microstructure at different cooling times

Figure 7 shows the room-temperature photoluminescence (PL) spectra of the single hexagonal ZnO microstructure after different retention times by micro-PL system. All measurements were performed under the same conditions with excitation wavelength of 325 nm, using He–Cd laser. The spectra consist of two emission bands, ultraviolet emission at about 385 nm (energy of 3.2 eV) and wide-band visible emission at from 450 to over 700 nm. For the hydrothermally grown ZnO microrods, the ultraviolet emission, also called near-band-edge (NBE) emission, originates from the radiative recombination of excitons [25] and the wide-band visible emission is due to the deep-level defect band in the ZnO band gap. The deep-level defects are the impurities and structural defects in the crystal such as oxygen and zinc interstitials or oxygen and zinc vacancies [26]. Besides, the inset of Fig. 7 shows UV emission spectra of the sample with 4-h retention. A satellite peak is observed at high energy near the normal UV emission spectra. The same phenomenon was observed by Q. D. Zhuang et al. [27] in PL spectra of InAsSb nanowires. The peak is also found in the other five samples with retention from 1–6 h. The UV emission spectra of those samples were fitted and the results are listed in Table 1. The first peak, PL peak 1, located at energy of 3.20 eV represents normal ultraviolet emission of ZnO grown by hydrothermal method. The second peak, PL peak 2, located at energy of 3.32 eV might be attributed to the morphology effect of the microrods; since the intensity ratio between satellite peak (peak2) and main peak (peak1) is small (0.158) in the sample without any retention and is growing with the samples that underwent self-etching process. The ratio has only little increase for the sample of 5-h retention. This might be due to the length of the sharp tip of the sample which is longer than the diameter of the laser spot. Therefore, the PL emission may not include the effect of the whole microrod top.

## Conclusions

ZnO microrods were synthesized on FTO substrates using zinc nitrate and hexamethylenetetramine by hydrothermal method. The as-grown microrods are hexagonal pillars with flat top end of (0001) surface. The top-end morphology of microrods can be changed by post retention at low temperature after hydrothermal growth. The top-end morphology changes from a flat to sharp tip and to a polygonal shape with 12 specific facets when the retention time changes from 0 to 1–5 and to 6 h, respectively. The morphology change is due to self-etching process that caused by the changing of pH value according to the temperature in the solution. The specific facets left were identified by Wulff construction of ZnO with [1$\overline{2}$10] directions as zone axis, since the direction points to the corner of top end which is the most vulnerable site for etching. Therefore, the 12 facets are the ($\overline{1}$014) and (10$\overline{1}$4) facets of each [1$\overline{2}$10] directions as zone axis. The morphology change also influences the PL spectra. A satellite peak in the UV emission spectra was observed. The peak may be attributed to the morphology effect of the microrods. The sharp tip on the top end of microrods may be applied to the field emission devices.

## Abbreviations

FESEM: field emission scanning electron microscopy
FTO: fluorine-doped tin oxide
HMT: hexamethylenetetramine
HRTEM: high-resolution TEM
MP: micropencil
MR: microrod
NBE: near band edge
ODS: octadecyltrimethoxysilane
PL: photoluminescence
SAED: selected area electron diffraction
TEM: transmission electron microscopy
UV: ultraviolet
XRD: X-ray diffraction
ZnO: zinc oxide
